# Mirror Neurons System Engagement in Late Adolescents and Adults While Viewing Emotional Gestures

**DOI:** 10.3389/fpsyg.2016.01099

**Published:** 2016-07-20

**Authors:** Emilie Salvia, Moritz Süß, Ruxandra Tivadar, Sarah Harkness, Marie-Hélène Grosbras

**Affiliations:** ^1^Laboratoire de Neurosciences Cognitives, UMR 7291, Centre National de la Recherche Scientifique and Aix-Marseille UniversitéMarseille, France; ^2^Centre National de la Recherche Scientifique, Fédération 3C (FR 3512), Aix-Marseille UniversitéMarseille, France; ^3^Centre for Cognitive Neuroimaging, Institute of Neuroscience and Psychology, University of GlasgowGlasgow, UK

**Keywords:** mirror neurons, TMS, motor evoked potentials, anger, late adolescence

## Abstract

Observing others’ actions enhances muscle-specific cortico-spinal excitability, reflecting putative mirror neurons activity. The exposure to emotional stimuli also modulates cortico-spinal excitability. We investigated how those two phenomena might interact when they are combined, i.e., while observing a gesture performed with an emotion, and whether they change during the transition between adolescence and adulthood, a period of social and brain maturation. We delivered single-pulse transcranial magnetic stimulation (TMS) over the hand area of the left primary motor cortex of 27 healthy adults and adolescents and recorded their right first dorsal interossus (FDI) muscle activity (i.e., motor evoked potential – MEP), while they viewed either videos of neutral or angry hand actions and facial expressions, or neutral objects as a control condition. We reproduced the motor resonance and the emotion effects – hand-actions and emotional stimuli induced greater cortico-spinal excitability than the faces/control condition and neutral videos, respectively. Moreover, the influence of emotion was present for faces but not for hand actions, indicating that the motor resonance and the emotion effects might be non-additive. While motor resonance was observed in both groups, the emotion effect was present only in adults and not in adolescents. We discuss the possible neural bases of these findings.

## Introduction

Previous studies have shown that visual information received during actions’ observation is also processed as motor information, i.e., in terms of a motor resonance. At the neural level this is implemented by the engagement of mirror neurons, mainly found in the premotor and parietal cortices, which are active both during action observation and during action execution. This mirror neurons system (MNS) might provide the foundation for social understanding. Indeed, mirroring external events would allow us to “resonate” with others while viewing them acting and might be crucial for understanding their intentions, beliefs and goals (Rizzolatti et al., 2001; Rizzolatti and Craighero, 2004; Fadiga et al., 2005; Agnew et al., 2007; Catmur et al., 2007). Understanding others’ intentions also requires emotional and empathic processing (Agnew et al., 2007). Interestingly, viewing others’ emotional facial expressions recruits brain regions involved while we ourselves experience similar emotions (e.g., Carr et al., 2003; Wicker et al., 2003). Furthermore, MNS activity has been shown to be higher in individuals showing higher accuracy in emotion discrimination and recognition (Enticott et al., 2008). The link between MNS and emotion is also highlighted by studies showing that exposure to emotional stimuli can modulate MNS response. For instance, Enticott et al. (2012a) have reported that the increased motor excitability during action observation was further enhanced if negative emotional stimuli had been presented before the observation period as compared to a condition where positive stimuli had been presented. Not only pictures or videos inducing emotions can affect movements but also linguistic material. Indeed, Spadacenta et al. (2014) showed decreased reaction time and mistakes in a reaching task after processing negatively valenced verbs as compared to neutral verbs, suggesting that the automatic attraction of attention by the former speeds up the activation of motor circuitry, illustrating the tight link between action and emotion even in language processing. Besides, a rich literature puts forward the engagement of the motor system during emotional processing (e.g., Frijda, 2009; Tamietto et al., 2009). In particular studies using transcranial magnetic stimulation (TMS) have revealed increased cortico-spinal excitability, reflecting activation of the motor system, while subjects were exposed to emotional stimuli unrelated to action, either in the visual (Baumgartner et al., 2007; Hajcak et al., 2007; Schutter et al., 2008; Coombes et al., 2009; Coelho et al., 2010; van Loon et al., 2010; Enticott et al., 2012a), auditory (Baumgartner et al., 2007; Komeilipoor et al., 2013) olfactory (Rossi et al., 2008), or verbal domain (Oathes et al., 2008; Baumert et al., 2011). Some of these studies reported larger motor evoked potentials (MEPs) only while viewing unpleasant stimuli as compared to neutral and pleasant ones (Coelho et al., 2010; Enticott et al., 2012a). Others found that, irrespective of valence, emotional arousal could enhance cortico-spinal excitability (Baumgartner et al., 2007; Hajcak et al., 2007; Coombes et al., 2009; van Loon et al., 2010; Borgomaneri et al., 2012). Lastly, while the tight link between action and emotion is undeniable, the direction of their mutual influence is not always well established. For example negative stimuli (e.g., painful, fearful stimulations) might either induce avoidance behaviors (Morrison et al., 2007; Schutter et al., 2008) or approaching ones (Avenanti et al., 2005). Nevertheless, altogether, these findings are in line with theories suggesting that emotionally salient stimuli might be viewed as motivators for action and thereby influence the execution of future movements (de Gelder, 2009; LeDoux and Damasio, 2013).

One question that remains unexplored is to which extent the “action-observation” and “emotion” effects on motor activity are comparable and whether they could add to each other. We set out to bring some elements of response to this question by combining emotional modulation and action observation and investigating the modulation of cortico-spinal excitability by emotions embedded into the observed action, in this case an angry gesture.

In addition, we wanted to explore how MNS activity in response to action observation and emotion perception could be modulated by individual factors related to social cognition. Some deficits in MNS functions have been reported in individuals with autism (Enticott et al., 2012b) pointing toward a relationship between MNS and social functioning, although the results are mixed (review in Hamilton, 2013). More convincingly, fMRI and TMS studies have reported a correlation between MNS activity and social abilities, such as empathy, in the normal population (Gazzola et al., 2006; Puzzo et al., 2009; Jola et al., 2012). If MNS activity is related to social cognition ability, one might expect to observe changes during the lifespan when social abilities develop. In this respect late adolescence is a crucial period when social behavior as well as brain structure and function undergo unique changes (Grosbras et al., 2007). We define the period of late adolescence operationally as the period comprising the transition between high school and higher education, thus ranging from about 17–19 years. Others have referred to this period as “emerging adulthood” (Arnett, 2000) or youth (Steinberg, 2013). This period is characterized by an abrupt change in social context, while fundamental social abilities, such as perspective taking (Dumontheil et al., 2010), theory of mind (Moor et al., 2012) and some aspects of emotion processing (Rothman and Nowiki, 2004; Tottenham et al., 2011) are still not adult-like. Brain activity during executive tasks involving social or emotional stimuli also changes in this age range (Monk et al., 2003; Hare et al., 2008; Veroude et al., 2013), suggesting that the increased sensitivity to emotional stimuli in late adolescents might be linked to immature connectivity between frontal and prefrontal regions supporting executive control and subcortical regions involved in emotional processing. In parallel, developmental morphometric brain imaging studies show that white matter structure and gray matter density display a protracted developmental time course into the early twenties, particularly in prefrontal, parietal and limbic regions important for linking executive control and emotion processing (Giedd and Rapoport, 2010; review in Lenroot and Giedd, 2006; Lebel and Beaulieu, 2011). In this view we hypothesize that the motor system and the MNS would be more influenced by emotion in late adolescents than in adults.

In summary, we asked two questions: (1) Is the MNS activity during action observation modulated by emotion embedded in the observed gesture? and (2) Does the emotion modulation of the MNS change with age, at the transition into adulthood?

To probe MNS activity, we used single-pulse TMS over the hand representation of the primary motor cortex while recording muscle activity. The resulting responses, the MEPs, provide us with a read-out of cortico-spinal excitability, and thus of motor system engagement, at the moment of stimulation. It is well established that MEP amplitude increases during action observation and that this reflects MNS activity (Fadiga et al., 1995; Strafella and Paus, 2000; Gangitano et al., 2001; Aziz-Zadeh et al., 2002; Maeda et al., 2002; Clark et al., 2003; Patuzzo et al., 2003; Montagna et al., 2005; Avenanti et al., 2007). We applied TMS while participants passively watched short videos of object-directed hand actions that were performed either in a neutral way or an in angry way. In accordance with previous studies using this method, the hand orientation in the video matched the one of the participant (Maeda et al., 2002), and we delivered the TMS pulse over the hemisphere contralateral (i.e., left) to the hand performing the action (i.e., right; Aziz-Zadeh et al., 2002), at the time of maximum use of the muscle where we recorded activity (Gangitano et al., 2001). Further, to look at the effect of emotion independently of the movement we also showed videos of facial expressions, either emotionally neutral or angry. We choose to use emotions of anger, which are perceived as a social threat signal (Green and Phillips, 2004), since their influence on motor responses has been most consistently reported (Schutter et al., 2008; Coelho et al., 2010; Enticott et al., 2012a; Ferri et al., 2013).

In line with the previous reports outlined above, we expected that both neutral hand action videos and angry face expression videos would induce larger MEP amplitudes than neutral face expression videos, which in turn should not be different from control videos of objects in motion. We also hypothesized that if the effects of emotion and action observation are independent we would observe a larger increase of the cortico-spinal excitability when they are combined in the angry hand action condition. In contrast, if they rely on the same modulatory pathway to the motor cortex, then their respective effect might not add. Furthermore, we hypothesized that the effect of emotion would be higher in late adolescents than in adults.

## Materials and Methods

### Participants

Healthy students from the University of Glasgow, Scotland, took part in this experiment. Forty-two datasets were recorded (20 adults and 22 adolescents). All datasets for which more than half of the MEP were contaminated by noise and thus undistinguishable or were inferior to 0.1 mV were discarded. Consequently, only 27 of them were included in this analysis: 14 young adults between 23 and 25 years old [mean (SD) age 24 (0.78), 9 females], 13 late adolescents between 17 and 19 years old [mean (SD) age 18 (0.82), 9 females]. Except two participants, all were right-handed. They were naïve to the aim of the study. They received ten British pounds as a compensation for their participation. Each participant filled a safety questionnaire ensuring that they had no contraindication to TMS (Rossi et al., 2009). A written informed consent was obtained from all the participants. This study was approved by the ethics committee of the College of Science and Engineering at the University of Glasgow (CSE01404) and in line with 1964 Declaration of Helsinki.

Thirty-two additional participants took part in an online experiment to rate the stimuli. They also belonged to two groups: 17 young adults between 22 and 27 years old [mean (SD) age 25 (1.41), 11 females], 15 late adolescents between 16 and 19 years old [mean (SD) age 18 (1.12), 9 females].

### Transcranial Magnetic Stimulation and EMG Recordings

Single-pulses of TMS were delivered over the left primary motor cortex (M1) using a circular coil (diameter 9 cm) connected to a biphasic MagStim Rapid2 Stimulator (Magstim, Whitland, UK). The maximum output on the single-pulse delivery mode is 3.5 Teslas.

The EMG activity of the first dorsal interossus (FDI) was recorded using a CED (Cambridge Electronic Device) amplifier and the software Signal (4.07). We placed three silver-chloride electrodes on the participants to record the right FDI activity. By palpating the FDI of the participants while they moved up and down the index finger, we put the active electrode on the belly of this muscle. The reference electrode was placed on the joint of the index finger and the ground electrode on the elbow. Prior to electrode attachment with surgical tape, all these sites were cleaned with alcohol. We also applied electrolyte cream (EC2 Astro-Med, Inc Subsidy) on the electrodes to improve the contact between the electrodes and the skin.

The first step in the TMS session consisted in localizing the optimal site on the scalp to evoke responses in the right FDI. Participants wore a tight swimming cap to allow us to mark localizations. They were seated comfortably with their head supported by padded chin- and forehead- rests. The coil was first placed over the approximate location of the hand motor area over the left hemisphere using vertex and inion landmarks (Clark et al., 2003). The stimulator output was set at 60% of the maximum. The target area was explored by displacing the coil in small steps until responses could be evoked in the right FDI and therefore MEPs detected. During this stage, isolated pulses were separated by at least 7 s. Once the spot with the highest responses was localized, we marked the site on the cap to ensure consistent coil positioning across the experiment. For the duration of the experiment, the same experimenter was responsible for holding the coil in place, with the help of an articulated arm (Manfrotto, Inc.) fixed on the same support as the chin- and forehead-rests.

The second step consisted in determining the resting motor threshold. To this end, we lowered the intensity stepwise until we reached an intensity for which we could detect only five MEPs larger than 50 mV out of 10 consecutive trials. This intensity was considered as the individual resting motor threshold. This threshold didn’t differ significantly between the two age groups [*t*(24) = 0.73, *p* = 0.47]: the means (SD) of the resting motor threshold were 45.43% (8.47) of the stimulator maximal output for adults and 47.61% (6.96) for adolescents. The intensity was set to 120% of the individual resting motor threshold for the experiment. Therefore, the means (SD) of the experiment stimulation intensities were 53.93% (9.63) for adults and 57.00% (8.26) for adolescents.

### Stimuli and Tasks (TMS and Online Experiment)

Five kinds of 2-s black-and-white video clips were presented on an 18-inch CRT screen located 45 cm away from the participants. They were selected from a set used in previous fMRI studies (Grosbras and Paus, 2006; Shaw et al., 2012; Tahmasebi et al., 2012). These stimuli showed hand actions (stirring with a spoon, cutting with scissors, picking up a phone, drawing with a pencil, moving a computer mouse, cutting with a knife, hammering and lifting a glass), face movements (e.g., actors twitched their nose, opened their mouth, blinked their eyes) or object movements (e.g., flag, metronome, water, wheel, helices or windscreen wipers). The hand and face movements could be performed either in a neutral or in an angry way. The object-directed hand action videos showed a right hand completing actions toward an object, with all actions clearly involving the right FDI. While participants watched these videos, the TMS pulse was timed to occur at the start of the grasp, which is likely to induce the highest motor facilitation (Urgesi et al., 2010). The face movements included either a shift from neutral to angry expressions or ambiguous non-emotional movements. Neutral object videos included videos of objects in movements without any human agent. This condition was included in order to verify the specificity of the action observation and emotion effects and was thus considered as the control condition. For the face and control stimuli, the timing of the TMS pulse was set so as to match, on average, that of the one determined for the hand action videos. There were eight different stimulus exemplars per condition, one being selected semi-randomly for each trial.

The videos were blocked per condition with a block comprising 24 videos separated by 5 s inter-trial intervals. Within a block, each individual stimulus was thus presented three times. One pulse per video was delivered. Two blocks were separated by 80 s long “rest” blocks during which a black-and-white picture of a tree was presented. During these “rest” blocks, we delivered 11 pulses with an inter-pulse interval of 7 s on average. The order of the five video blocks (Hand Neutral: HN, Hand Angry: HA, Face Angry: FA, Face Neutral: FN, Control: Ctr) was counterbalanced across participants.

Participants were instructed to look carefully at the videos (or at the tree during the rest blocks), while staying, as much as possible, still and relaxed. To prevent voluntary contractions of the right FDI, they placed their right hand on a pillow and were asked to relax their forearm and hand. Because of the strong noise of the TMS, earplugs were offered to the participants. The whole experiment lasted ∼25–30 min.

Thirty-two independent participants performed an online experiment in order to assess the subjective judgment of the stimuli. They were requested to rate the emotion perceived, while they viewed the videos used in the TMS experiment (hand actions, face movements and objects movements). They rated each video using a 15-points Likert scale: from “*strong happiness*” (-7) to “*strong anger*” (7) going through “*medium happiness*,” “*neutral*” (0) and “*medium anger.*” The happiness option was added to ensure that the neutral videos did not convey any emotional activation and that the angry videos had a negative valence. Each video was presented only once. The presentation order was counterbalanced across participants.

### Data Analysis

#### Stimuli Ratings

The participants had to rate the intensity of the stimuli according to two categories, happiness and anger, by using a single scale from -7 (happiness) to 7 (anger) going through 0 (stimuli considered as neutral). We investigated the effect of both the different categories of videos (five types of videos: HN, HA, FA, FN, Ctr) and the age-group (adults vs. adolescents) on the ratings using a 5 × 2 repeated-measures ANOVA. *Post hoc* pairwise comparisons were performed using *t*-tests. The critical *p*-value was set at 0.05 for all the analyses.

#### TMS Data

We measured the peak-to-peak MEP amplitudes (in mV) using an in-house Matlab script. The MEPs onsets varied across participants (between 17 and 32 ms) but were constant within participant. Therefore, we specified, for each participant, an interval of interest in which the peak-to-peak amplitudes were extracted automatically. In our dataset all trials had a clearly distinguishable MEP superior to 0.1 mV. For each video block and each rest block, we computed the median amplitude across trials. A repeated-measures ANOVA conducted on the median amplitudes of the five rest conditions revealed a trend toward a decrease in MEPs values between the beginning and the end of the experiment [*F*(4,26) = 2.12, *p* < 0.08 η^2^ = 0.70], with no significant group differences in median amplitude [*F*(1,25) = 1.48, *p* = 0.24, η^2^ = 0.05] nor interaction between group and change across session [*F*(1,4) = 1.58, *p* = 0.34, η^2^ = 0.04]. ANOVAs conducted in individuals on the trials of the five different rest conditions revealed significant (*p* < 0.05) changes in 16/27 participants (7/13 adolescents). Therefore, we decided to normalize the median value of each video block by dividing it by the median value of the rest block presented just before.

We entered these data into a 2 × 5 mixed-model repeated-measure ANOVA, with group (adults vs. adolescents) as a between subjects variable, condition (HN, HA, FA, FN, Ctr) as the within subject factor and subject and condition/subject as random intercepts and random slopes (Scheffé, 1959, chapter 8; Barr et al., 2013). To explore the interactions, we then specified the following contrasts to test our hypotheses. To test whether emotion and action had an additive effect on cortico-spinal excitability we compared HA and HN, with age group as a between subject factor and subject/condition as a random effect factor. To investigate how age affects the action observation effect and its specificity we compared HN and Ctr as well as HN vs. FN between the two groups, still modeling subject/condition as random slope. To investigate how age affects the emotion effect we tested group differences in the contrasts FA vs. Ctr and FA vs. FN. We present results without any correction for multiple comparisons. This statistical analysis was conducted in R (R-2015 package; R Core Team, 2015).

## Results

### Behavioral Results

**FIGURE 1:**
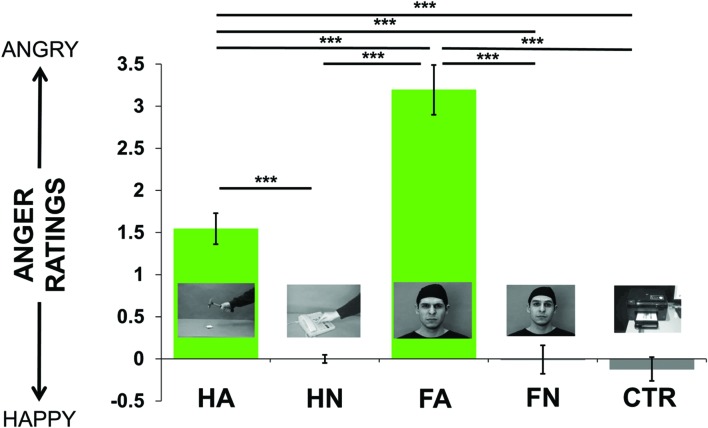
**Mean of the ratings’ values across the group (from -7 = happiness to 7 = anger, going through 0 = neutral) for each kind of videos (i.e., Hand Anger, Hand Neutral, Face Anger, Face Neutral, Control).** *Post hoc* pairwise *t*-tests were performed to statistically quantify the *a priori* differences it may exist between each condition. ^∗∗∗^*p* < 0.001, corrected for multiple comparisons.

All stimuli were perceived as either neutral or angry and none was perceived as happy. The 5 (video conditions) × 2 (age groups) repeated-measures ANOVA showed a main effect of videos on value of ratings [*F*(4,120) = 69.92, *p* < 0.0001], with no age effect [*F*(1,30) = 0.37, *p* = 0.5], nor interaction [*F*(4,120) = 0.83, *p* = 0.5]. *T*-tests were performed to statistically quantify the differences it may exist between pairs of conditions (see **Figure 1**). As expected, HA and FA were both perceived as inducing a significantly greater anger intensity than HN [HA/HN: *t*(31) = 8.29, *p* < 0.0001; FA/HN: *t*(31) = 10.65, *p* < 0.0001], Ctr [HA/Ctr: *t*(31) = 8.53, *p* < 0.0001; FA/Ctr: *t*(31) = 11.44, *p* < 0.0001] and FN [HA/FN: *t*(31) = 6.67, *p* < 0.0001; FA/FN: *t*(31) = 9.47, *p* < 0.0001]; therefore confirming the previous validation of our stimuli. We also observed that angry face stimuli (FA) were rated as showing significantly greater anger intensity than angry hand stimuli [FA/HA: *t*(31) = 6.58, *p* < 0.0001]. All these reported comparisons were corrected with Bonferroni correction. We didn’t observe any difference between neutral videos: neutral hand, face and control were rated similarly. Importantly, when looking at individual ratings within each category (i.e., HN, HA, FA, FN, Ctr), we did not observe any difference between the eight different video exemplars.

### TMS Results

**FIGURE 2:**
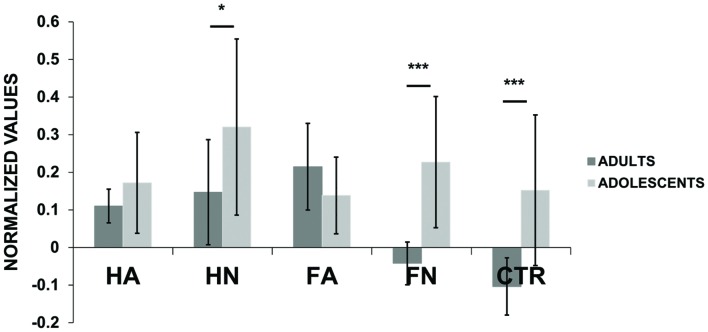
**Motor evoked potential (MEP) amplitudes (normalized values) for each kind of videos (i.e., Hand Anger, Hand Neutral, Face Anger, Face Neutral, Control) for adults and adolescents.** *Post hoc* pairwise *t*-tests were performed to statistically quantify, for each condition, the differences it may exist between adults and adolescents. ^∗^*p* < 0.05, ^∗∗∗^*p* < 0.001. Bars indicate the standard error.

**Figure 2** presents the normalized MEPs averaged for adults and adolescents separately. Qualitatively, we observed the expected pattern in adults’ results, reflecting both the motor resonance effect (i.e., neutral hand actions produced higher excitability than control and neutral faces) as well as the emotion effect (i.e., angry faces induced higher excitability than control and neutral ones; see **Figure 2** and **Table 1**). In contrast late adolescents displayed a different and less expected pattern, with high MEPs for HN but not for emotional stimuli (see **Figure 2** and **Table 1**). This figure also shows that adolescents present higher cortico-spinal excitability than adults while viewing HN [*F*(1,646) = 5.576, *p* = 0.0185], FN [*F*(1,646) = 21.67, *p* < 4 × 10^-6^] and Ctr [*F*(1,633) = 17.32, *p* = 4 × 10^-5^] videos while no difference was observed when participants viewed emotional stimuli (FA: *p* = 0.232; HA: *p* = 0.235).

**Table 1:** Results of the *post hoc* tests which, assess the differences between conditions for adults (left) and adolescents (right) separately.

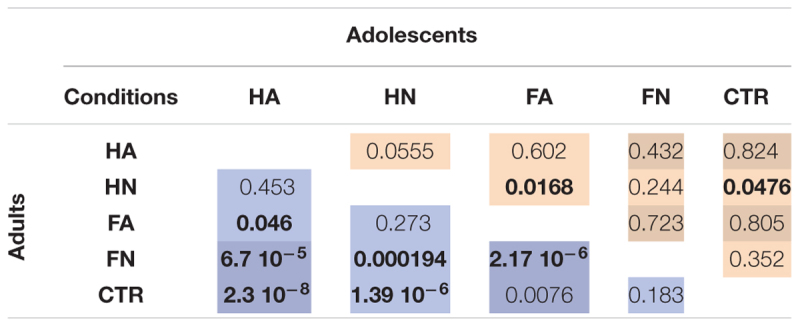

*Bold numbers highlight significant differences between conditions. Hatched cells highlight the significant interactions (i.e., video condition × group).*

The ANOVA of the normalized MEP amplitudes revealed a main effect of video type [*F*(4,3211) = 8.86, *p* = 4. 10^-7^], a main effect of group [*F*(1,3211) = 7.79, *p* = 0.0053] and an interaction between group and video condition [*F*(4,3211) = 2.743, *p* = 0.027]. We computed *post hoc* planed contrasts to explore these main effects and interactions based on our hypotheses. They revealed that, as expected, neutral hand (HN) induced significantly higher excitability compared to control stimuli [*F*(1,1277) = 4.068, *p* = 0.041] reflecting the action observation effect. Neutral hand also induced higher excitability than neutral face stimuli [*F*(1,1290) = 7.93, *p* = 0.0049], reflecting the specificity of this effect. There was no interaction between these effects and age group. We also observed an effect of emotion: the condition FA (emotion effect without action observation effect) induced higher change in excitability than control [*F*(1,1276) = 11.66, *p* < 7 × 10^-5^] and FN [*F*(1,1291) = 4.418, *p* = 0.0357]. The FA vs. Ctr contrast showed a mild interaction with age group [*F*(1,1276) = 3.471, *p* = 0.062]. Indeed comparing FA and Ctr tested in each group separately showed a significant effect in the adult group (*p* < 0.0076) but not in the late adolescent group (*p* < 0.805). The contrast FA vs. FN also showed an interaction with age group [*F*(1,1291) = 15.970, *p* < 7 × 10^-5^]. Indeed comparing FA and FN tested in each group separately showed also a significant effect in the adult group (*p* < 2.17 × 10^-6^) but not in the late adolescent group (*p* < 0.723).

We used additional planned contrasts to compare the effect of action observation and emotion, respectively, on cortico-spinal excitability and their interaction with age. There was no difference between hand with and without emotion [HA vs. HN; *F*(1,1292) = 0.513, *p* = 0.47], indicating that observing a gesture performed with anger does not add to change in excitability due to neutral action observation. No interaction with age group was observed. To directly compare the magnitude of the effects of action and emotion we compared directly FA vs. HN. We did not observe any difference [*F*(1,1293) = 1.135, *p* = 0.287]. For completes, **Table 1** presents all the possible *post hoc* tests performed in the two age groups separately.

In summary, our data show a main effect of condition, which replicates the action observation and emotion effect reported in the literature. They indicate that the action observation and the emotion effects on cortical excitability are of similar magnitude and not additive in the case of angry hand action. Our data also shows that the video conditions influence cortico-spinal excitability differently in the two age groups. While the action observation effect is present in both groups, the emotion effect is significant only for the adults and not for the late adolescents. This interaction is mainly due to increased excitability for the control and the neutral face conditions, showing high responses variability, in the adolescent group.

## Discussion

We used single-pulse TMS to assess the influence of action observation only, emotion only, and the combination of both of these components on cortico-spinal excitability. We also tested whether the effects of emotion and action observation on motor resonance change with age, at the transition between adolescence and adulthood.

Using dynamic stimuli – i.e., videos presenting neutral/angry others’ hand actions, neutral/angry facial expressions, or neutral moving objects – we confirmed that both action perception and emotional stimuli facilitate cortico-spinal excitability. Combining these two factors by presenting angry gestures did not show any additional augmentation in motor excitability compared to the action observation (neutral hand movements) or the emotion (angry face movements) factors presented in isolation. Lastly, while the action observation effect was present in all participants, the effect of emotion was observed in adults but not in late adolescents, although the interaction was marginally significant (*p* < 0.06).

### Motor Resonance Modulation by Action Observation and Emotion

By showing significantly larger changes in MEPs while participants viewed neutral hand action videos compared to neutral objects or neutral face expressions, our data complement the corpus of experiments showing that motor excitability is increased during action observation (Fadiga et al., 1995; Strafella and Paus, 2000; Gangitano et al., 2001; Aziz-Zadeh et al., 2002; Maeda et al., 2002; Clark et al., 2003; Patuzzo et al., 2003; Montagna et al., 2005; Urgesi et al., 2006, 2010; Avenanti et al., 2007, 2012).

The higher MEPs observed for angry faces compared to control stimuli or to neutral faces also replicates previous studies reporting that viewing emotional stimuli increases cortico-spinal excitability in a task-unrelated way. These studies, however, had mostly used pictures from the International Affective Picture System (IAPS, Baumgartner et al., 2007; Hajcak et al., 2007; Coombes et al., 2009; Coelho et al., 2010; van Loon et al., 2010; Borgomaneri et al., 2012, 2014; Enticott et al., 2012a). Only a couple of studies had shown that presentation of social emotional signals such as body movements (Borgomaneri et al., 2012, 2015) or fearful faces (Schutter et al., 2004) also yielded increase in MEPs amplitude. Taken together these and our results support a close relationship between the mechanisms involved in processing threat-related signals and the motor system. At the neural level this could be supported by anatomical connections between limbic regions, such as the amygdala, and cortical motor, premotor and sensorimotor areas (e.g., Grèzes et al., 2014). This is in line with, functional studies showing activations in both motor- (premotor and inferior frontal cortices) and emotion- (amygdala, insula) related regions while participants watch emotional facial expressions (e.g., Carr et al., 2003, reviewed in Grèzes and Dezecache, 2014).

Our main goal here was to compare the effects that action observation and emotion, respectively, have on cortico-spinal excitability and how these may interact in a condition where the emotion is conveyed by the observed action. We revealed that the difference in MEPs between emotional and neutral stimuli was observed only in the context of face stimuli, which were not directly related to the targeted muscle representation. In contrast, in the action observation condition, we did not observe any difference between the neutral and angry hand actions. In addition, the effect of emotion for the face stimuli was of same amplitude as the effect of action observation, with no significant difference between the angry face condition and either of the hand conditions. Therefore, while combining action perception and emotion in a single stimulus, we didn’t observe larger motor responses than when only one of these components was included in a stimulus, indicating that the two effects are not additive in this case.

This might be explained by a ceiling effect: after a certain point is reached, all muscle fibers are recruited and the motor response cannot increase anymore. However, although we did not build recruitment curves, this seems unlikely when stimulating at 120% of individual motor threshold (Kojima et al., 2013; Goetz et al., 2014). The maximum amplitude we observed across all participants was 6 mV with average maximum amplitude being 2.9 mV (*SD* = 1.55) across participants, which is below the maximum that could be expected in most participants for the FDI. Alternatively, the emotion induced by angry hand movements might not have been intense enough compared to the emotion conveyed by angry face expressions, and thus too weak to induce an effect above the action observation effect. Yet ratings showed that the difference between angry and neutral hand movements was highly significant despite no effect at the level of the MEPs. This seems thus to rule out the possibility that the lack of physiological difference between HA and FA was due to the weaker conscious emotional perception.

The lack of difference between emotion and action observation here is at odd with a previous study, which showed indication of additivity between increased cortico-spinal excitability due to emotional arousal and increased excitability due to preparation of movement. van Loon et al. (2010) measured MEPs elicited by M1 TMS during motor preparation in a reaction-time task. Prior to and irrelevant to the task, pictures that could bear a positive, negative or neutral valence were presented. The presentation of emotional pictures was associated with larger MEPs than the presentation of neutral ones. Crucially, this effect was larger during the motor preparation phase when the cortico-spinal excitability was the highest. The discrepancy between this report and our results suggests that hand-action observation might influence motor excitability through a different pathway than motor preparation, possibly overlapping with the mechanism by which emotion influences the motor system (see discussion in Bestmann and Krakauer, 2015). This is in line with the report by Oliveri et al. (2003) showing that pre-conditioning TMS applied over the supplementary motor area (SMA) selectively increased motor excitability during responses to emotional stimuli, but not for responses to neutral stimuli or at rest. In contrast pre-conditioning the dorsal premotor cortex did not show this effect. This suggests that a specific pathway including the SMA, which may be modulated by amygdala activity (de Gelder et al., 2012; Grèzes et al., 2014), is involved in transforming motivation into motor responses and that this pathway could be influenced by the emotional state as well as by the observation of other people. Modulatory changes occurring in this pathway could add to changes occurring at the level of motor programming, as it was the case in van Loon et al. (2010) study discussed above and in studies showing increased motor activity during imitation as compared to action observation or execution alone (Clark et al., 2003) or studies showing additive effects of action observation and motor imagery (which supposedly engages motor preparation; Sakamoto et al., 2009).

### Age Effect: Emotions Effect on Motor Resonance Is Observed in Adults but Not in Adolescents

As late adolescence is a period of increased emotional sensitivity we could have expected an increased motor resonance while viewing emotional stimuli. If anything, we observed a trend toward a weaker effect of emotion in adolescents and when we considered the adolescent group alone, we did not observe differences in motor response amplitudes when viewing emotional compared to neutral videos. The lack of emotion effect in adolescents is likely not explained by the way adolescents felt the emotion expressed by the videos as no group differences were observed regarding the ratings. It might be due to large responses for FN and Ctr videos, greater than those observed in adults. However, this might be lessened as high responses variability is observed in this population for neutral faces and control stimuli. Therefore, to explain adolescent group pattern, more studies may be needed. Another explanation might be that the angry faces were processed differently in adolescents and in adults. FMRI studies have shown that adolescents engaged the same brain regions as adults, yet with lower activity for the angry faces but not for the neutral ones (Shaw et al., 2012; Tahmasebi et al., 2012). Other studies have reported higher amygdala activity in adolescents than in adults while viewing social threat-related stimuli (Hare and Casey, 2005; Guyer et al., 2008), although late adolescents have rarely been tested. Also, there might be less integration between emotion and motor control neural circuits in adolescents. Indeed, (1) a few studies have shown that connections between the limbic system (including the amygdala) and the prefrontal cortex mature from childhood to adulthood and contribute to developmental differences in the efficient recruitment of cognitive control (Elzinga and Bremner, 2002; Liston et al., 2006; Steinberg, 2008; Christakou et al., 2011) and (2) a direct pathway between the amygdala and motor cortical regions including the primary motor cortex, the premotor cortex and the supplementary motor area was also recently described by Grèzes et al. (2014), which could provide an anatomical basis for the influence of motor behavior by amygdala activity. An immaturity of these limbic/motor connections could account for the lack of emotion effect on motor excitability in adolescents. Although these results should be taken with care due to the low sample sizes, they highlight the importance of studying late adolescence as a period of changes in integration between action and emotion representation. They call for further brain imaging studies investigating this question.

## Author Contributions

SH and M-HG designed the study; ES, RT, MS, and SH performed the study; MS and ES analyzed the data; ES and M-HG wrote the paper.

## Conflict of Interest Statement

The authors declare that the research was conducted in the absence of any commercial or financial relationships that could be construed as a potential conflict of interest.
